# *In Vitro* and *In Vivo* Antitumor Activity of [Pt(*O*,*O′*-acac)(γ-acac)(DMS)] in Malignant Pleural Mesothelioma

**DOI:** 10.1371/journal.pone.0165154

**Published:** 2016-11-02

**Authors:** Antonella Muscella, Carla Vetrugno, Luca Giulio Cossa, Giovanna Antonaci, Francesco De Nuccio, Sandra Angelica De Pascali, Francesco Paolo Fanizzi, Santo Marsigliante

**Affiliations:** 1 Laboratory of Cell Pathology, Department of Biological and Environmental Sciences and Technologies (Di.S.Te.B.A.), University of Salento, Lecce, Italy; 2 Laboratory of Cell Physiology Di.S.Te.B.A., University of Salento, Lecce, Italy; 3 Laboratory of Human Anatomy and Neuroscience, Di.S.Te.B.A., University of Salento, Lecce, Italy; 4 Laboratory of Inorganic Chemistry, Di.S.Te.B.A., University of Salento, Lecce, Italy; Institute of Biochemistry and Biotechnology, TAIWAN

## Abstract

Malignant pleural mesothelioma (MPM) is an aggressive malignancy highly resistant to chemotherapy. There is an urgent need for effective therapy inasmuch as resistance, intrinsic and acquired, to conventional therapies is common. Among Pt(II) antitumor drugs, [Pt(*O*,*O′*-acac)(γ-acac)(DMS)] (Ptac2S) has recently attracted considerable attention due to its strong *in vitro* and *in vivo* antiproliferative activity and reduced toxicity. The purpose of this study was to examine the efficacy of Ptac2S treatment in MPM. We employed the ZL55 human mesothelioma cell line *in vitro* and in a murine xenograft model *in vivo*, to test the antitumor activity of Ptac2S. Cytotoxicity assays and Western blottings of different apoptosis and survival proteins were thus performed. Ptac2S increases MPM cell death in vitro and in vivo compared with cisplatin. Ptac2S was more efficacious than cisplatin also in inducing apoptosis characterized by: (a) mitochondria depolarization, (b) increase of bax expression and its cytosol-to-mitochondria translocation and decrease of Bcl-2 expression, (c) activation of caspase-7 and -9. Ptac2S activated full-length PKC-δ and generated a PKC-δ fragment. Full-length PKC-δ translocated to the nucleus and membrane, whilst PKC-δ fragment concentrated to mitochondria. Ptac2S was also responsible for the PKC-ε activation that provoked phosphorylation of p38. Both PKC-δ and PKC-ε inhibition (by PKC–siRNA) reduced the apoptotic death of ZL55 cells. Altogether, our results confirm that Ptac2S is a promising therapeutic agent for malignant mesothelioma, providing a solid starting point for its validation as a suitable candidate for further pharmacological testing.

## Introduction

Malignant pleural mesothelioma (MPM) is a very aggressive cancer of the pleura. MPM is a clinical challenge because its incidence increases, and is expected to rise further due to the widespread use of asbestos in diverse developing nations [1]. The most effective treatment proven to prolong life of malignant mesothelioma patients is the combination of multi-folate inhibitors, pemetrexed or raltitrexed and cisplatin (cis-diamminedichloridoplatinum(II) or CDDP), but still the median survival is only 12 months, and response rates are approximately 40% [2].

Since almost half of patients are primary resistant and finally almost all develop resistance with biological bases not yet clarified, there is an urgent need for an effective therapy. Thus, attention was paid to the design of new platinum compounds with stronger pharmacological properties, less toxicity and more favourable therapeutic indices equated to CDDP. In this context, [Pt(O,O′-acac)(γ-acac)(DMS)] (Ptac2S) [3, 4], a platinum drug for non genomic targets, has recently gained increasing attention as potential anticancer agent. This thanks to its high and selective cytotoxicity towards cancer, as observed in immortalized cell lines and confirmed in breast cancer cells in primary cultures [5–10] and *in vivo* [11, 12]. Remarkably, in a preclinical model based on the subcutaneous injection of MCF-7 breast cancer, Ptac2S stands out for higher anticancer activity than CDDP toward both the murine tumor models examined, and also for an enhanced in vivo pharmacokinetics (PK), biodistribution and tolerability in Wistar rats. PK studies with Ptac2S revealed prolonged Pt persistence in systemic blood circulation and decreased nephrotoxicity and hepatotoxicity, two major target sites of CDDP toxicity. Theoretically, this compound could provide a broader spectrum of application since, alongside to the cytotoxic effects, Ptac2S also exerts specific anti-metastatic responses *in vitro* [13, 14].

Keeping in mind these results it is important to understand whether or not Ptac2S is cytotoxic to malignant mesothelioma, which is resistant to conventional therapies. The 3 major histologic types of malignant mesothelioma are sarcomatous, epithelial, and mixed. Being epithelial type the most represented [15], here we used the human ZL55 cells, obtained from asbestos-exposed patients [16] that represent a trustworthy model for evaluating the ability of Ptac2S to cause cell death and also to understand its mechanisms of toxicity.

Previously we have shown CDDP was cytotoxic for ZL55 cells with an IC50 obtained after 48 h of 11 μM. This cytotoxicity was due to the triggering of apoptosis and to the activation of two isoforms of PKCs (delta and alpha), with opposing roles: a pro-apoptotic role of the PKC-δ and instead an antiapoptotic role of the PKCα.

In the current investigation, we aimed to assess the cytotoxicity of Ptac2S both *in vitro* and *in vivo* and to compare to that of CDDP, and also to understand what differences we may have in the cellular mechanisms that determine the ZL55 death/survival fate.

## Materials and Methods

### Cell culture

The human mesothelioma cell line ZL55 [15] was grown in RPMI 1640 medium (Sigma-Aldrich, St. Louis, MO, USA) supplemented with 10% fetal bovine serum (FBS), penicillin (100 U/ml) and streptomycin (100mg/ml). The cells were maintained at 37°C in the presence of 5% CO_2_ in air. Cells were grown to 70–80% confluence and then treated with CDDP and Ptac2S at various concentrations and for different incubation periods.

### *In vivo* xenograft experiments

Athymic Nude mice (6 wk old, female, 20 to 30 g body weight) were purchased from Harlan Laboratories (San Pietro al Natisone UD, Italy) and maintained under pathogen-free conditions. They were given free access to standard food and water, with a 12 h light-dark cycle at a temperature of 22+/−2°C. Approximately 6 x 10^6^ ZL55 cells were injected subcutaneously in the right flank. Tumor size was measured with slide callipers and volumes were calculated as (LxW^2^)/2, where L and W are the major and minor diameters, respectively. Once tumor volumes reached ~200 mm^3^, mice were randomly divided into four groups, in such a manner as to minimize weight and tumor size differences among the groups. Mice were treated by a single intravenous injection of saline as a control, or two doses (5 and 10 mg/kg) of Ptac2S, or 10 mg/kg CDDP.

Both the research team and the veterinary staff monitored animals twice daily. Health was monitored by weight (twice weekly), food and water intake, and general assessment of animal activity, panting, and fur condition. As described previously [11], all animals received care in compliance with the Principles of Laboratory Animal Care formulated by the National Society for Medical Research and the Guide for the Care and Use of Laboratory Animals prepared by the Institute of Laboratory Animal Resources, published by the National Institutes of Health (NIH Publication No. 86–23, revised 1985), as well as in accordance with the Italian laws on animal experimentation (art. 4 and 5 of D.L. 116/92). Ethical Committee on Animal Research (Ministero della Salute D.M. 109/2014-B) approved the protocols. All efforts were made to minimize suffering to animals; thus, the experimental procedures used in the work described in this article were in compliance with the guidelines for reporting experiments involving animals [17]. All injections or surgical procedures were performed using sterile technique with efforts made to minimize trauma to the animals. The maximum size the tumors allowed to grow in the mice before euthanasia was 2000 mm^3^. After 35 days of treatment, animals were anesthetized with a mixture of 1.75% isofluorane/air and were sacrificed through cervical dislocation. Excised tumors were divided and either fast frozen in liquid nitrogen or placed in a paraformaldehyde solution, (4%) and 20 h later it was placed in 70% ethanol until paraffin inclusion.

### Cytotoxicity assay

We evaluated the IC_50_ in ZL55 cells with SRB and MTT assays. The SRB (sulforhodamine B) assay and the conversion of MTT (3-(4,5-dimethylthiazol-2-yl)-2,5-diphenol tetrazolium bromide) by mesothelioma cells were used as an indicator of cell number as described previously [6]. The percentage of survival was calculated as the absorbance ratio of treated to untreated cells. Viable cells were also counted by the trypan blue exclusion assay and light microscopy. The data presented are means ± standard deviation (S.D.) from eight replicate wells per microtitre plate.

### Clonogenic survival assay

Cells were seeded in 100 mm Petri dishes at low density (~3X10^4^ per dish) and left to adhere for 24 h in a standard medium. Crescent concentrations of Ptac2S or CDDP were added; after 2 h cells were washed, immediately treated with trypsin, resuspended in single-cell suspension, and plated for the determination of macroscopic colony formation. After 15 days of growth, colonies were fixed with a 3:1 mixture of methanol/acetic acid and stained with crystal violet. Only colonies consisting of more than 50 cells were scored. Four separate experiments were performed using duplicate samples.

### Cell cycle analysis

Cell cycle analysis was performed using a FACSCanto flow cytometer (Becton-Dickinson, CA, USA). After the indicated treatments, cells were washed with cold PBS and harvested by centrifugation. Then, cells were re-suspended in 70% (v/v) cold ethanol and stored at– 20°C overnight. After 30-minute incubation with propidium iodide solution in the dark, cell cycle distribution was analyzed by flow cytometry cell sorting. Cell cycle distribution (sub-G1, G0/G1, S and G2/M phase fraction) was analyzed by using FlowJo software (Ver. 7.6.5, TreeStar, USA).

### Apoptosis analysis

For 4,6-diammine-2-phenylindol (DAPI) staining, cells treated with CDDP or Ptac2S were fixed with 3% formalin and stained with 1mg/ml DAPI in PBS for 10 min. Cells were mounted on glass slides, covered, and analysed using fluorescence microscopy. For statistical analysis of each experiment, 5–10 fields (magnification 400X) were counted (between 400 and 700 cells in total). The mean ± S.D. was calculated and displayed as bar graph.

### Spectroscopic analysis of mitochondrial membrane depolarization

Mitochondrial membrane depolarization was detected by a shift in fluorescence emission of the lipophilic cationic probe 5,5’,6,6’-tetrachloro-1,1’,3,3’-tetraethylbenzimidazolo-carbocyanine iodide (JC-1), as described previously [18].

### Preparation of subcellular fraction and Western Blotting analysis

Preparation of subcellular fraction, western blotting analysis and immunodetection were performed as previously reported [19]. The purity of fractions was tested by immunoblotting with anti a subunit of Na^+^/K^+^-ATPase monoclonal antibody (membrane protein), anti-histone-3/4 polyclonal antibody (nuclear proteins), β-actin (cytoplasmic protein) or porin (mitochondrial membrane protein). The blots were stripped and used for sequential incubation with control antibodies. Densitometric analysis was carried out on the Western blots using the NIH Image (v1.63) software (National Institutes of Health, Bethesda, MD, USA). The pixel intensity for each region was analysed, the background was subtracted and the protein expressions were normalized to β-actin loading control for each lane.

### Design and preparation of small interfering RNA (siRNA)

siRNAs were prepared by an in vitro transcription method, according to the manufacturer’s protocol (Promega, Madison, WI, USA). Initially, four siRNA target sites specific to human PKC-δ and PKC-ε, as determined by blast analysis, were chosen. For each siRNA, sense and antisense templates were designed based on each target sequence and partial T7 promoter sequence. The ZL55 cells were transfected with siRNA duplexes using the protocol supplied with the CodeBreaker siRNA transfection reagent (Promega Corporation) as described previously [7].

### siRNA transfection

The cells (50–70% confluence) were transfected with siRNA duplexes using the protocol supplied with the CodeBreaker siRNA transfection reagent (Promega, Madison, WI, USA). Briefly, the transfection reagent was first diluted into RPMI medium without serum and antibiotics for about 15 min, and then the sense and non-sense siRNA (siRNA-NS) duplex were added to the medium to form a lipid-siRNA complex. Following additional 15 min incubation, transfection was initiated by adding the lipid-siRNA complex to 6-well plates. The final concentrations of siRNAs were 10 nM. Immunoblottings were performed 24 and 48 h post-transfection to determine the efficiency of siRNA incorporation in cells and to measure the protein expressions. Quantitative analysis of protein expression, as measured by intensity of immunoreactivity in siRNA transfected cells, revealed a very high reduction in PKC-ε and PKC-δ expression.

### Materials

Ptac2S was prepared according to previously reported procedures [4, 5]. CDDP was purchased from Sigma-Aldrich, Chemicals (Milan, Italy). RPMI 1640 medium, antibiotics, glutamine and foetal bovine serum were purchased from Celbio (Milan, Italy). Caspase -9, -7 and -3, Bax, Bcl-2, poly(ADP-ribose) polymerase-1 (PARP-1), were obtained from Cell Signalling Technology (Celbio, Milan, Italy). PKC isoforms antibodies, phospho-specific p-38MAPK and total (phosphorylated and unphosphorylated) p-38MAPK antibodies, anti-porin (i.e. anti-voltage-dependent anion selective channel 1, VDAC1), goat anti-rabbit conjugated with peroxidase, as well as control antibodies were obtained from Santa Cruz Biotechnology (Santa Cruz, CA, USA). All others reagents were from Sigma-Aldrich (Milan, Italy).

### Statistical analysis

The experimenter measuring the tumours and the data analyst were unaware of the treatments given to the animals. Data, presented as means ± SD, were collected in blinded fashion and analysed using GRAPHPAD PRISM 5 software (GraphPad Software, La J olla, C A, USA). Unpaired Student’s t-test, the Mann-Whitney U test or one-way ANOVA, and when this returned P < 0.05, post hoc analysis using Bonferroni test were performed; we used the Bonferroni-Dunn post hoc test in the ANOVA after a significant omnibus F-test. P < 0.05 was accepted as a level of statistical significance.

## Results

### Cytotoxicity of Ptac2S

Alteration of cell viability and induction of apoptosis along with cell cycle arrest were investigated in ZL55 cells after treatment with Ptac2S or CDDP.

The cytotoxicity *in vitro* data shown here were obtained by MTT metabolic assay and confirmed by SRB assay to rule out potential effects of Ptac2S on mitochondrial enzymes. Indeed, comparable results were obtained when cell number was directly determined by cell counting (data not shown); consequently, we used SRB assay in the experiments herein reported.

**Fig 1:**
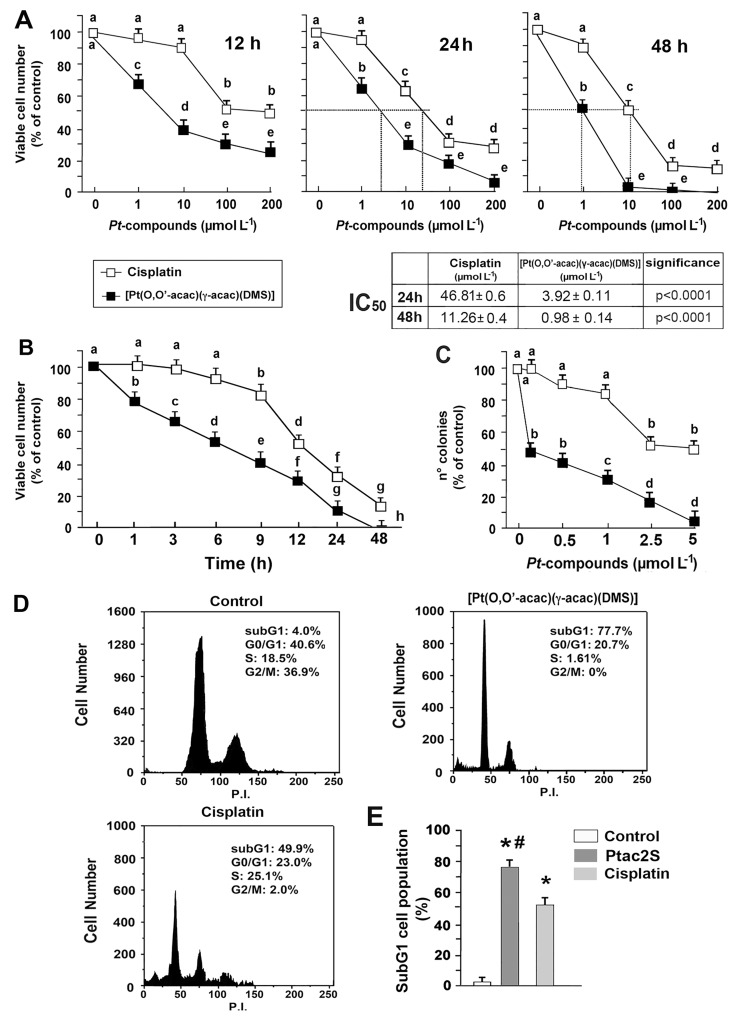
The sensitivity of ZL55 cells to Ptac2S and cisplatin. **(A)** Cells were treated with or without increasing concentration of Ptac2S or CDDP for 12, 24 and 48 h; **(B)** or were continuously exposed to 100 μM CDDP or Ptac2S. Cell viability was monitored by SRB assay and data are presented as means ± S.D. of six-independent experiments with eight replicates in each, and are presented as percent of control. **(C)** Clonogenic survival assay in cells treated with the indicated amounts of Ptac2S or CDDP for 2 h, and after 15 days of growth, colonies consisting of more than 50 cells were scored. The percentage of number colonies represents the means ± S.D. of six-independent experiments. Values with shared letters are not significantly different according to Bonferroni/Dunn post hoc tests. **(D)** After the treatment with 5 μM Ptac2S or 50 μM CDDP for 24 hours, cell cycle profiling was performed by FACSCanto flow cytometer as described under Materials and methods. Representative FACS histogram from six separate experiments is shown. **(E)** Comparison of sub-G1 DNA content in Ptac2S or CDDP treated cells. *P < 0.01, significantly different from saline control; #P < 0.01, significantly different between Ptac2S and CDDP. **Inset**: The IC_50_ values to CDDP and Ptac2S calculated after 24 and 48 h.

Incubation of ZL55 cells with CDDP and with Ptac2S (1–200 μM) provoked the dose-dependent inhibition of cell survival (Fig 1A). One hundred μM Ptac2S killed about 50% cells after 7 h of treatment only, while 100 μM CDDP provoked the same effect after 18 h (Fig 1B). Ptac2S was significantly more cytotoxic than CDDP also by using the clonogenic assay (Fig 1C).

Ptac2S has shown cytotoxicity approximately 12-fold greater than that noted for CDDP (IC_50_ at 24 h were 3.9 ± 0.11 μM for Ptac2S and 46.8 ± 0.6 μM for CDDP, n = 6) (see table in Fig 1). Thus, in subsequent experiments performed in order to compare the pathways bringing to cell death, we chose to use 5 μM Ptac2S and 50 μM CDDP.

Flow cytometric analysis also showed a significant accumulation of cells in sub-G1 phase after treatment with the Ptac2S or CDDP, for 24 h (Fig 1D); in agreement with results in Fig 1A, a significant difference in the sub-G1 phase cell number was observed between Ptac2S and CDDP (p<0.01, Fig 1D). Moreover, G2/M cell cycle arrest along with a significant decrease in the number of cells in both G0/G1 and S phase was observed simultaneously (Fig 1D).

### Ptac2S causes caspases proteolysis, enhances pro-apoptotic Bax protein and reduces anti-apoptotic Bcl-2 protein

**Fig 2:**
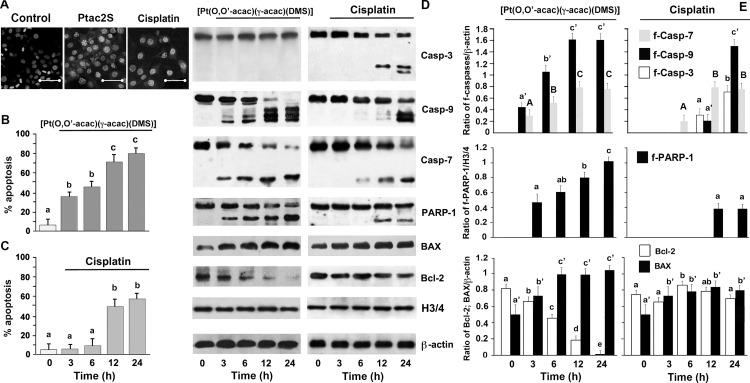
Ptac2S and CDDP provoked apoptosis. **(A, B, C)** ZL55 cells treated or not with 5 μM Ptac2S or 50 μM CDDP for the indicated times were stained with DAPI. **(A)** The representative fields of one of five independent experiments after 24 hours of incubation are shown. Scale bar = 50 μm.The quantification of the percentage of apoptotic nuclei obtained from cells stained with DAPI (mean ± S.D.; n = 5) and incubated with Ptac2S (**B**) or CDDP (**C**). Values with shared letters are not significantly different according to Bonferroni/Dunn post hoc tests. **(D)** Cytosolic and nuclear proteins were obtained from ZL55 cells, treated or not with 5 μM Ptac2S (right) or 50 μM CDDP (left). Samples were dissolved in SDS buffer and separated on SDS gel. Immunoblotting was performed using monoclonal antibodies specific to PARP, caspases-3, -7 and -9, Bax and Bcl2. Sequential incubation with anti-actin confirmed the equal protein loading. These results are representative of six independent experiments. **(E)** Densitometic analysis of Bcl-2 and BAX or active caspase-3 (f-Casp 3), active caspase-7 (f-Casp 7), active caspase-9 (f-Casp 9) and fragmented PARP-1 (f-PARP) normalized to β-actin and fragmented PARP (f-PARP) normalized to H3/4 of experiments shown in (D). The data are means ± S.D. of fIve different experiments. P < 0.0001 by one-way ANOVA for Bcl-2, f-caspases and f-PARP (n = 5); P < 0.001 by one-way ANOVA for BAX (n = 5); values with shared capital and lower case letters are not significantly different according to Bonferroni/Dunn post hoc tests.

Fig 2A shows the results of a DAPI staining. The time course of the nuclear changes revealed that ZL55 cells reacted differently from platinum compounds, inasmuch as 50% of apoptotic cells were seen after 6 h of treatment with Ptac2S (Fig 2B) but after 12 h of treatment with CDDP (Fig 2C).

The cleavage patterns of caspase-3, -7, -9 and PARP-1 were analysed by western blotting. Both Ptac2S and CDDP caused proteolytic activation of caspase-7 and -9, but the effects of Ptac2S were faster (Fig 2D). Whilst CDDP also provoked the proteolytic activation of caspase-3, the Ptac2S-treated cells did not show such activation, at least up to 24 h of treatment (Fig 2D). As shown in Fig 2D, PARP-1 was cleaved both in cells treated with 5 μM Ptac2S that with 50 μM CDDP; however, proteolysis was faster with Ptac2S. Sequential incubation of the blots with an antibody against actin showed that the amount of protein loaded was the same.

Caspase-9 is related to mitochondrial apoptotic pathway with Bcl-2 family of proteins regulating the mitochondrial permeability. Therefore, we also assessed the effects of 5 μM Ptac2S and 50 μM CDDP on Bax and Bcl-2 proteins expression using western blot analysis in whole cells. Ptac2S increased Bax expression whilst decreased the levels of Bcl-2. The effects of CDDP on Bax were slighter and were absent those on Bcl-2 (Fig 2D).

### Ptac2S causes mitochondrial membrane depolarization and release of mitochondrial cytochrome-c

**Fig 3:**
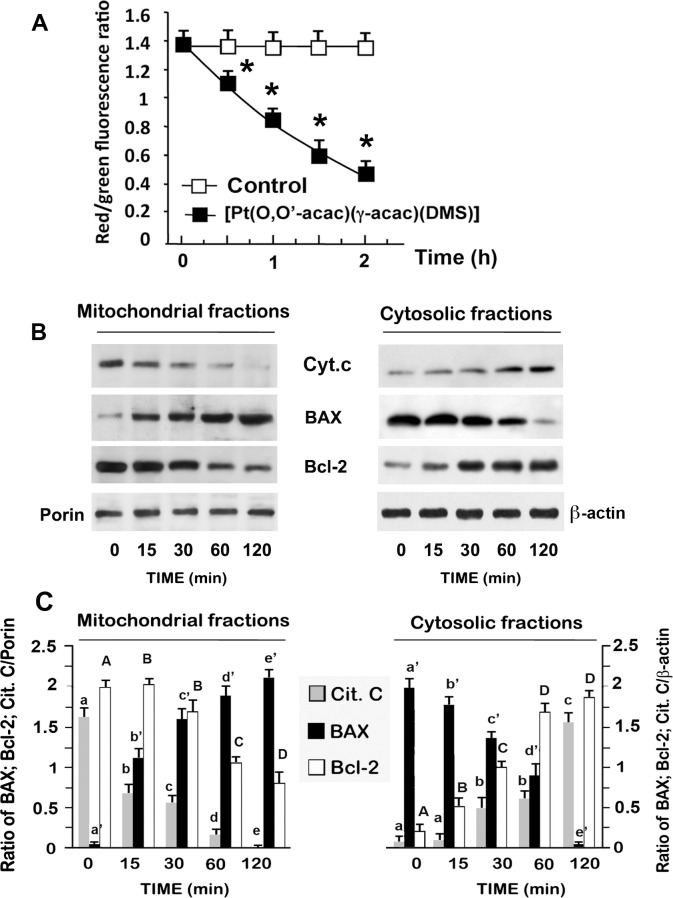
Effect of Ptac2S on ΔΨm and apoptotic proteins in ZL55 cells. **(A)** Fluorescent spectra of JC-1 in ZL55 cells treated or not with 5 μM Ptac2S for the indicated time. The data are means ± S.D. of six different experiments and are presented as red J-aggregates/green monomer JC-1 fluorescence ratio. Asterisks indicate values that are significantly different (p < 0.05) from control at the same time point. **(B)** Mitochondrial and cytosolic fractions were prepared at the indicated times of Ptac2S treatment (5 μM), and the kinetics of Bax and Bcl-2 cytosol-to-mitochondria translocations and the release of mitochondrial cytochrome c were examined by western blotting. Porin and β-actin served as a mitochondrial and cytosolic indicators, respectively. The Fig is representative of six independent experiments. **(C)** Densitometic analysis of cytochrome c, Bcl-2 and Bax, normalized to β-actin (cytosolic fractions) or to porin (mitochondrial fractions), of experiments shown in (B). The data are means ± S.D. of six different experiments and are presented as the ratio of the densitometric values between Bcl-2 or BAX and β-actin/porin. P < 0.0001 by one-way ANOVA for all (n = 6); values with shared capital and lower case letters are not significantly different according to Bonferroni/Dunn post hoc tests.

Ptac2S also provoked the cytosol-to-mitochondria translocation of Bax, and the translocation of Bcl-2 from mitochondria to cytosol, phenomena that precede the decrease in the electric potential of the mitochondrial membrane (ΔΨm) (Fig 3A and 3B).

A decrease in ΔΨm accompanies early apoptosis in many systems. ΔΨm was monitored by fluorescence of the cationic lipophilic dye JC-1. After addition of 5 μM Ptac2S, ΔΨm decreased slowly, as determined by mean aggregate fluorescence of JC-1 (Fig 3A). After 40 min of Ptac2S incubation, ΔΨm was completely annulled. Five μM of carbonylcyanide-m-chloro-phenylhydrazone (a depolarizing agent), incubated for 10 min, was considered as the positive control (data not shown).

Since cytochrome c release is associated to the induction of apoptosis, we evaluated the effect of 5 μM Ptac2S on cytochrome-c release from mitochondria. Mitochondrial and cytosolic fractions were separated as described in materials and methods and cytochrome c was assessed by immunoblot analysis. Cytosol from untreated cells did not show detectable cytochrome c protein. The level of the mitochondrial cytochrome c declined significantly after 15–60 min of treatment with Ptac2S (Fig 3B).

#### Activation of PKC-δ is critical for the Ptac2S-induced apoptosis in ZL55 cells

**Fig 4:**
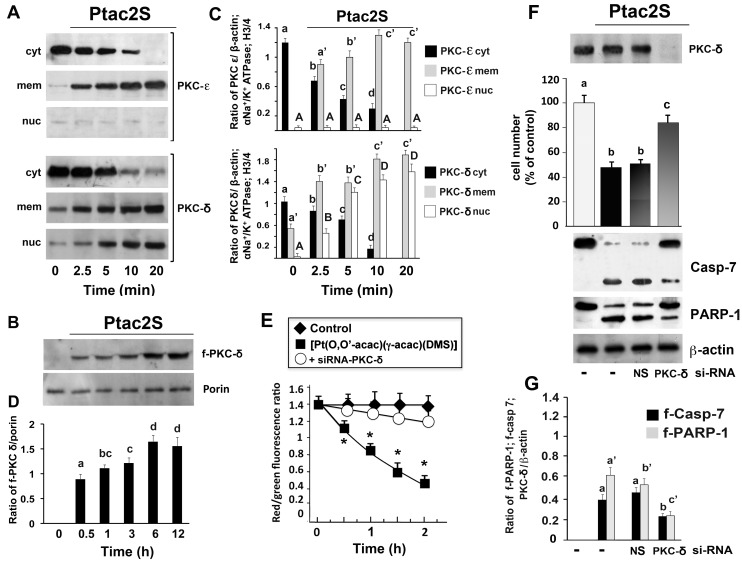
PKC-δ induces apoptosis in ZL55 cells. ZL55 cells were treated without or with 5 μM Ptac2S for the indicated times. For PKCs translocation studies, cytosol (cyt), membrane (mem), nuclei (nuc) **(A)** and mitochondrial **(B)** fractions were analysed by Western blotting with specific antibodies. The Figs are representative of six independent experiments. **(C and D)** The data of densitometic analysis are means ± S.D. of six different experiments and are presented as the ratio of the densitometric values between PKCs and β-actin (cytosolic fractions) or α-subunit of Na+/K+ATPase (membrane fractions), or H3/4 (nuclear fractions) or porin (mitochondrial fractions). P < 0.0001 by one-way ANOVA for all (n = 6); values with shared letters are not significantly different according to Bonferroni/Dunn post hoc tests. **(E)** Cells were transfected with siRNA–PKC-δ and then were incubated or not with Ptac2S for the indicated times. The data are means ± S.D. of six different fluorescent spectra of JC-1 presented as red J-aggregates/green monomer JC-1 fluorescence ratio. Asterisks indicate values that are significantly different (p < 0.05) from control at the same concentration and time point. **(F)** Cells were transfected with siRNA–PKC-δ or control siRNA (NS) and then were incubated with Ptac2S. Viable cell number was determined 24 h later by SRB assay. The data are means ± S.D. of six different experiments run in eight replicates and are presented as percent of control; values with shared letters are not significantly different according to Bonferroni/Dunn post hoc tests. Western blotting of total lysates was performed with specific anti-PKC-δ or with anti-caspase-7 or anti-PARP-1 antibodies. The Fig is representative of six independent experiments. **(G)** Densitometic analysis of f-caspase-9 (f-Casp-9) and f-PARP-1, normalized to β-actin are shown. The data are means ± S.D. of five different experiments. P < 0.0001 by one-way ANOVA for all (n = 5); values with shared letters are not significantly different according to Bonferroni/Dunn post hoc tests.

As the cellular effects of Ptac2S accompany the activation of various PKC isoforms, we here have studied their possible activation. Since activated PKCs move to cell membranes from the cytosol, the distribution of PKCs in ZL55 cells incubated with 5 μM Ptac2S (from 0 to 20 min) was analyzed using immunoblotting. Between the isoforms expressed by cells ZL55, only two, the PKC-ε and the PKC-δ, showed a cytosol-to-membrane translocation; PKC-δ also translocated to the nuclear fraction (Fig 4A). Similarly to what happened with CDDP [18], the cells treated with Ptac2S also show the proteolytic activation of PKC-δ. While the full-length PKC-δ moved to the membrane and nuclei, its fragment was located to the mitochondria (Fig 4B and 4D). In contrast to CDDP, the PKC-α was not activated (data not shown).

The role of PKC-δ was verified using small interfering RNA. The inhibition of PKC-δ blocked the effects of Ptac2S on the mithocondrial ΔΨm (Fig 4E) and the survival of Ptac2S-treated ZL55 cells (Fig 4F). Preparatory experiments by Western blotting showed that PKC-δ-siRNA reduced PKC-δ expression and that non-specific siRNA (siRNA-NS) had no silencing effect (Fig 4F upper). Furthermore, PKC-δ–siRNA also inhibited the Ptac2S-provoked caspase-7 activation and PARP-1 cleavage (Fig 4F and 4G). Thus, the role of activated PKC-δ appears the same as the role it plays when the cells are incubated with CDDP [18].

### Ptac2S induces MAPKs phosphorylation

**Fig 5:**
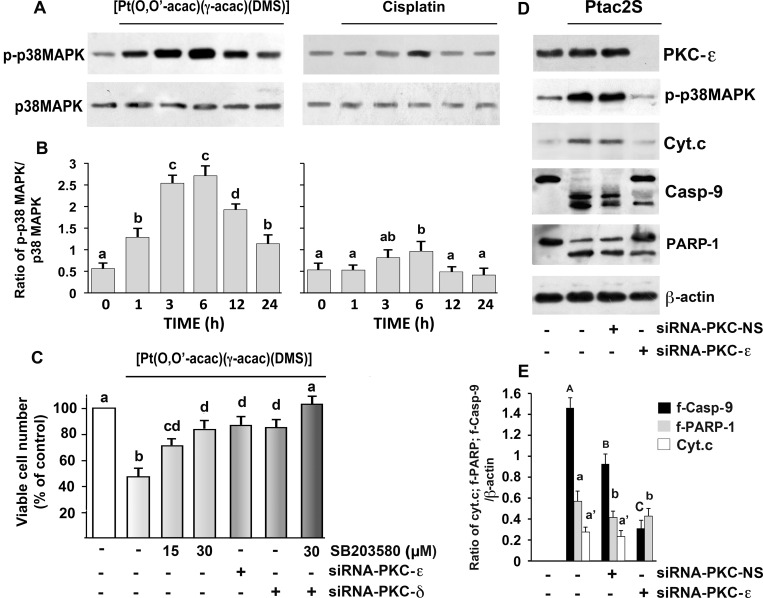
Role of PKC-ε in Ptac2S-induced apoptosis in ZL55 cells. (**A**) Cells were treated or not with 5 μM Ptac2S or with 50 μM CDDP for indicated time. Cell lysates were analysed by Western blotting with anti-unphosphorylated and phosphorylated p38MAPK antibodies. (**B**) Densitometic analysis of p-p38MAPK normalized to p38MAPK. The data are means ± S.D. of five different experiments. Values with shared letters are not significantly different according to Bonferroni/Dunn post hoc tests. (**C**) Cells were transfected with siRNA–PKC-δ or siRNA–PKC-ε or pretreated with SB203580 (15, 30 μM) and then incubated with Ptac2S; viable cell number was determined 24 h later by SRB assay. The data are means ± S.D. of five different experiments run in eight replicates and are presented as percent of control; values with shared letters are not significantly different according to Bonferroni/Dunn post hoc tests. (**D**) Cells were transfected with siRNA–PKC-ε or control siRNA (NS) and then were incubated with Ptac2S. Cytosolic or nuclear (for PARP-1) fractions were analysed by Western blotting with antibodies against PKC-ε, phosphorylated p38MAPK, cytochrome c (Cyt. c), caspase-9 (Casp-9) and PARP-1; β-actin was used as a control for protein loading. Representative immunoblots of five experiments are depicted. (**E**) Densitometic analysis of f-caspase-9 (f-Casp-9), f-PARP-1 and cytochrome c (Cyt. c), normalized to β-actin are shown. The data are means ± S.D. of five different experiments. P < 0.0001 by one-way ANOVA for all (n = 5); values with shared letters are not significantly different according to Bonferroni/Dunn post hoc tests.

We have previously demonstrated that Ptac2S activates the MAPK signaling pathway in several tumor cell lines [5, 7, 8, 14]. In the specific case of ZL-55, we have also shown that CDDP causes activation of the MAPK ERK1/2 [18]. In accordance with previous studies, Western blot analysis using specific antibodies to phosphorylated p38MAPK, revealed that Ptac2S provoked their phosphorylation, without altering the global level, as uncover by antibodies against un-phosphorylated form (Fig 5A).

### The p38MAPK inhibitor SB203580, significantly reduced Ptac2S-induced cytotoxicity (Fig 5C)

To investigate whether or not PKCs were involved in Ptac2S-mediated p38MAPK activation, we used molecular (PKC-ε–siRNA and PKC-δ–siRNA) techniques, in order to specifically inhibit PKC-ε and PKC-δ, and establish their role in p38MAPK control. Preliminary experiments by Western blotting demonstrated that PKC-ε–siRNA and PKC-δ–siRNA were able to reduce PKC-ε and PKC-δ expressions, and that non-specific siRNA (siRNA-NS) had no silencing effect on PKC-ε and PKC-δ expressions (Fig 4F and Fig 5D). The PKC-ε–siRNA (10 nM) inhibited Ptac2S- induced p38MAPK phosphorylation (Fig 5D), and promoted the survival of Ptac2S-treated ZL55 cells (Fig 5C) and also inhibited the Ptac2S-provoked caspase-9 activation, PARP-1 cleavage and the release of cytochrome c into the cytoplasm (Fig 5D and 5E).

### Antitumor Activity of Ptac2S in a Preclinical Model of MPM

To test the *in vivo* efficacy of Ptac2S, we employed a preclinical model based on the subcutaneous injection of the ZL55 MPM cells in the flank of BALB/c nude mice. Once the tumours had reached the size of ~50 mm^3^, the mice were randomly divided into five groups in such a way as to keep down weight and tumor size differences between the groups. 10 mg/kg of Ptac2S was previously found to be efficacious without significant side effects in animal studies involving xenografts of human breast cancer cells [11]. Accordingly, afterwards the administration of a single intravenous injection of saline as a control, or 10 mg/kg of Ptac2S or 10 mg/kg of CDDP, we evaluated, by a vernier calliper, the volumes of the tumours every three days for 5 weeks.

**Fig 6:**
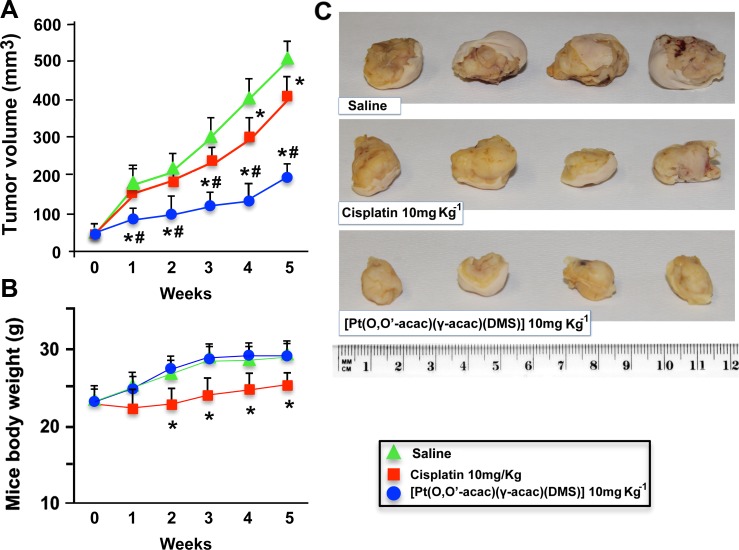
Growth inhibitory effect of Ptac2S and CDDP *in vitro* and in a xenograft model of Mesothelioma. Balb/c nude mice carrying mesothelioma developed by injection of ZL55 cells (around 50 mm^3^) received intravenous Ptac2S (10 mg/kg) or CDDP (10 mg/kg); tumour volume **(A)** and body weight of mice **(B)** were measured every 3 days for 35 days in total. The results are presented as mean ± S.D. (animals per group n = 8). *P < 0.05, significantly different from saline control; #P < 0.05, significantly different between Ptac2S and CDDP. **(C)** After killing, the tumors were collected and measured.

We then calculated for each group the mean volume of tumors and finally drew the relative growth curves. During the 30 days, the average tumour volume augmented from 52.5 ± 17.22 to 498.42 ± 43.97 mm^3^ for the saline group, 431.45 ± 50.57 mm^3^ for the CDDP group (10 mg/kg; p>0.05), 188.72 ± 32.53 mm^3^ for the Ptac2S group (Fig 6A).

Mice inoculated with ZL55 cells showed a statistically significant reduction of tumor volume at every time point in the Ptac2S groups compared with both not treated and CDDP-treated mice (*p* < 0.05; Fig 6A).

From treatment initiation until tumor resection, no significant loss of mice body weight was noticed in mice receiving 10 mg/kg Ptac2S (Fig 6B). In addition, throughout the course of the observation period, no damage in health was seen and the overall behaviour was not dissimilar from that of untreated animals.

## Discussion

Chemotherapic agents used in standard treatment of MPM show poor activity and also Pt-based drugs has limited efficacy. At present, only the combination of CDDP plus pemetrexed has improved clinical outcome in a phase III trial that yielded a response rate of 41% and survival benefits, when compared to single agent therapies, although the outcome was still extremely unsatisfactory showing an overall survival time of about 12 months [2, 20, 21]. Since there are no known biological bases of why about half of the MPM patients are primarily resistant and all finally develop resistance [22] there is an urgent need for effective therapy.

Using ZL55 mesothelioma cells, derived from an epithelial tumour biopsy of the pleural cavity of a 52-year old male, prior to treatment, with a known history of exposure to asbestos [23], we previously studied the cytotoxic effects of CDDP and highlighted the roles of PKC-δ and PKC-α: whilst PKC-δ is a pivotal portion of the apoptotic program, PKC-α mediates pro-survival effects [18]. In this paper we used a different Pt-based compound (Ptac2S) in order to determine its cytotoxicity and to unravel the cellular mechanisms, seeking comparisons with the previous study i.e. focusing on the roles of PKC and MAPK in the determination of death or survival. Ptac2S is a Pt(II) complex containing two acetylacetonate (acac) ligands and dimethylsulphide (DMS) in the metal coordination sphere that has shown interesting biological activities [5–12]. Differently from CDDP, for which the activity appears to be genomic, formation of DNA adducts, and non-genomic [5, 6], Ptac2S shows a small reactivity with nucleobases and a characteristic reactivity with sulphur ligands, indicating that the cellular targets could be protein amino acid residues. This can make it inherently less capable of evoking chemoresistance [6].

First of all we report here the different dynamics of the effects of Ptac2S on cell apoptosis compared to CDDP: the cytotoxic effects of Ptac2S were faster than those elicited by CDDP.

The proteolytic enzymes, caspases, have a critical role in the execution of apoptosis. It is suggested that CDDP may induce apoptosis through both caspase-3-dependent and independent pathways [24, 25]. Both caspase-3 and -7 can cleave the ubiquitous nuclear enzyme PARP-1 (poly(ADP-ribose) polymerase-1), a unique sensor of DNA breaks [26]. In our experiments, PARP-1 cleavage was detected after 3 h of Ptac2S treatment, in accordance with the rapid apoptosis onset. Usually, when cells undergo apoptosis, the 32 kDa pro-enzyme caspase-3 is proteolytically transformed into the active heterodimeric complexes composed of a 20 kDa (p20) and an 11 kDa (p11) subunit [27, 28]. In our study, these p20 and p11 subunits were detected in CDDP—but not in Ptac2S-treated cells, as observed in other studies [5–9]. Caspase-7 cleavage pattern was detected earlier in Ptac2S-treated cells compared to CDDP treatment, and matched the cleavage of PARP-1, thus supporting the study showing that caspase-7 is responsible for PARP-1 cleavage [29].

Consistently with previous results, in Ptac2S-treated ZL55 cells, the procaspase-9 activation proceeded together with the generation of the mature caspase-7 form, indicating the involvement of the intrinsic pathway. Ptac2S more rapidly than CDDP dissipated mitochondrial membrane potential (ΔΨm). Such depolarization is accompanied by the release of cytochrome c into the cytoplasm, activation of caspase-9 and subsequent activation of the executioner caspases to orchestrate apoptosis. The apoptosis associated to mitochondrial events induced by Ptac2S is facilitated by the altered expression (Bcl-2 down-regulation) and the mitochondrial localization (translocation of Bax) of apoptotic regulators [5–9].

The down-regulation of Bcl-2 protein amount noted in various models of apoptosis diminishes its ability to form heterodimers with the relative pro-apoptotic protein Bax, or to link with specific molecules preventing its antiapoptotic activity [30, 31]. In contrast, in mesothelioma cell lines and MPM cancer samples, the protective character of Bcl-2 is less evident [32, 33]. Albeit in some mesothelioma cell lines, low levels of Bcl-2 mRNA and protein were found, treatment with oligonucleotides Bcl-2 antisense lowered the apoptosis threshold in ZL34 mesothelioma cells [34]. Besides caspases, it has been demonstrated that specific protein kinases, comprehending PKC and MAPK families, also mediated apoptosis [8, 35–37].

In fact, we show here and in a previous paper, PKC-δ appears to be a crucial element in the pathway linking both Pt(II) compounds to apoptosis [18]. In the present paper we also show that Ptac2S-activated PKC-ε is responsible for the sustained activation of MAPK p38. As stated above, activated MAPKs can be components of the apoptotic program [38] and indeed we showed previously that p38 and JNK contributes to the apoptosis provoked by Ptac2S in breast cancer and in SH-SY5Y human neuroblastoma cells [7, 8]. Also in this paper we demonstrate that administration of Ptac2S to ZL55 causes activation of MAPKs and that p38 has a pro-apoptotic role, while neither ERK nor JNK seem implicated in this process. The role of p38 is underscored by the fact that its inhibition significantly reduced Ptac2S-induced cytotoxicity. Similarly, active p38 is required for the apoptosis in leukaemia cells [39], in U-937 promonocytic cells treated with cadmium chloride [40] and in mouse cortical neuronal cells treated with calyculin A, a selective inhibitor of Ser/Thr phosphatase I and IIA [41].

Although further studies are needed to identify the specific intracellular targets of p38, it is known that the activation of MAPKs might suppress the surviving function of Bcl-2, 38–40 and might explain why mesothelioma cells undergo Ptac2S-triggered apoptosis despite expressing high levels of Bcl-2. In fact, it has been suggested that one of the important reasons able to confer to mesothelioma its strong resistance to chemotherapy is the overexpression of anti-apoptotic proteins of the Bcl-2 family (such as Bcl-xL and Mcl-1 protein) [42]. Our in vitro findings suggest that Ptac2S might prove to be a more effective drug than CDDP in the treatment of MPM. The good results obtained in vitro have encouraged us to prepare a study on mice in vivo. Thus, we assessed the antitumor effects of Ptac2S in a xenograft model of mesothelioma developed by injection of ZL55, and compared with the effects obtained with CDDP. *In vivo* antitumor activity was consistent with the *in vitro* sensitivity, since mice inoculated with ZL55 cells showed a statistically significant reduction of tumor volume at every time point in the Ptac2S groups compared with both not treated and CDDP -treated mice. The difference of the effects *in vivo* between Ptac2S and CDDP was considerable inasmuch as Ptac2S induced up to 62% reduction of tumor mass compared to an average 10% inhibition obtained with CDDP. We previously showed pharmacokinetics studies with Ptac2S that revealed prolonged Pt persistence in systemic blood circulation, reduced neurotoxicity, nephrotoxicity and hepatotoxicity and decreased acute toxicity compared to CDDP [11].

In conclusion, whilst the data obtained are consistent with recent results obtained in ZL55 cells with CDDP [23] we show that these cells are much more sensitive to Ptac2S that more efficiently induces apoptosis and greatly reduces the tumor xenografts in mice. Concerning the intracellular events that Ptac2S causes in ZL55 cells, it is noteworthy the fact that, compared to CDDP, there is activation of PKCε, while remains the fundamental pro apoptotic role of PKC-δ. In addition, the lack of the antiapoptotic action of PKCα is probably one of the factors behind the increased toxicity of Ptac2S compared to CDDP in ZL55 cells.

## Abbreviations

CDDP: cisplatin
FBS: fetal bovine serum
MPM: malignant pleural mesothelioma
MTT: 3-(4,5-dimethylthiazol-2-yl)-2,5-diphenol tetrazolium bromide
PK: pharmacokinetics
SRB: sulforhodamine B
ΔΨm: electric potential of the mitochondrial membrane

## References

[1] Delgermaa V, Takahashi K, Park EK, Le GV, Hara T, Saraha T. Global mesothelioma deaths reported to the World Health Organization between 1994 and 2008, Bull. World Health Organ. 89 (2011) 716–724, 724A–724C. 10.2471/BLT.11.086678 22084509 PMC3209980

[2] Vogelzang NJ, Rusthoven JJ, Symanowski J, Denham C, Kaukel E, Ruffie P et al, Phase III study of pemetrexed in combination with cisplatin versus cisplatin alone in patients with malignant pleural mesothelioma, J. Clin. Oncol. 21 (2003) 2636–2644. 10.1200/JCO.2003.11.136 12860938

[3] De Pascali SA, Papadia P, Ciccarese A, Pacifico C, Fanizzi FP, First examples of b–diketonate platinum(II) complexes with sulfoxide ligands, Eur. J. Inorg. Chem. 5 (2005) 788–796.

[4] De Pascali SA, P. Papadia P, Capoccia S, Marchiò L, Lanfranchi M, Ciccarese A et al, Hard/soft selectivity in ligand substitution reactions of b–diketonate platinum(II) complexes, Dalton Trans. 37 (2009) 7786–7795. 10.1039/b909209a 19759954

[5] Muscella A, Calabriso N, De Pascali SA, Urso L, Ciccarese A, Fanizzi FP, et al, New platinum(II) complexes containing both an O,O'–chelated acetylacetonate ligand and a sulfur ligand in the platinum coordination sphere induce apoptosis in HeLa cervical carcinoma cells, Biochem. Pharmacol. 74 (2007) 28–40. 10.1016/j.bcp.2007.03.027 17481588

[6] Muscella A, Calabriso N, Fanizzi FP, De Pascali SA, Urso L, Ciccarese A, et al, [Pt(*O*,*O′*–acac)(γ–acac)(DMS)], a new Pt compound exerting fast cytotoxicity in MCF–7 breast cancer cells via the mitochondrial apoptotic pathway, Br. J. Pharmacol. 153 (2008) 34–49. 10.1038/sj.bjp.0707576 18026127 PMC2199379

[7] Muscella A, Calabriso N, Vetrugno C, Fanizzi FP, De Pascali SA, Marsigliante S, The signalling axis mediating neuronal apoptosis in response to [Pt(O,O'–acac)(γ–acac)(DMS)], Biochem. Pharmacol. 81 (2011) 1271–1285. 10.1016/j.bcp.2011.03.007 21420390

[8] Muscella A, Vetrugno C, Fanizzi FP, Manca C, De Pascali SA, Marsigliante S, A new platinum(II) compound anticancer drug candidate with selective cytotoxicity for breast cancer cells, Cell Death Dis. 4 (2013) e796 10.1038/cddis.2013.315 24030148 PMC3789173

[9] Vetrugno C, Muscella A, Fanizzi FP, Cossa LG, Migoni D, De Pascali SA, et al, Different apoptotic effects of [Pt(O,O'–acac)(γ–acac)(DMS)] and cisplatin on normal and cancerous human epithelial breast cells in primary culture, Br. J. Pharmacol. 171 (2014) 5139–5153. 10.1111/bph.12831 24990093 PMC4253461

[10] Grimaldi M, Santin G, Insolia V, Dal Bo V, Piccolini VM, Veneroni P, et al, [Pt(O,O'-acac)(γ-acac)(DMS)] versus cisplatin: apoptotic effects in B50 neuroblastoma cells, Histochem. Cell Biol. 145 (2016) 587–601. 10.1007/s00418-015-1396-1 26748644

[11] Muscella A, Vetrugno C, Migoni D, Biagioni F, Fanizzi FP, Fornai F et al (2014) Antitumor activity of [Pt(O,O'–acac)(γ–acac)(DMS)] in mouse xenograft model of breast cancer. Cell Death Dis 5: e1014 10.1038/cddis.2013.554 24457958 PMC4040677

[12] Muscella A, Vetrugno C, Migoni D, Biagioni F, Calabriso N, Calierno MT et al (2016) Antitumor and Antiangiogenic Activities of [Pt(O,O’-acac)(γ-acac)(DMS)] in a xenograft model of human renal cell carcinoma. Br J Pharmacol 173 (2016) 2633–2644. 10.1111/bph.13543 27351124 PMC4978158

[13] Muscella A, Calabriso N, Vetrugno C, Urso L, Fanizzi FP et al (2010). Sublethal concentrations of the platinum(II) complex [Pt(O,O'–acac)(γ–acac)(DMS)] alter the motility and induce anoikis in MCF–7 cells. Br J Pharmacol 160: 1362–1377. 10.1111/j.1476-5381.2010.00782.x 20590627 PMC2938808

[14] Muscella A, Vetrugno C, Calabriso N, Cossa LG, De Pascali SA, Fanizzi FP, et al (2014b) [Pt(O,O'-acac)(γ-acac)(DMS)] alters SH-SY5Y cell migration and invasion by the inhibition of Na+/H+ exchanger isoform 1 occurring through a PKC-ε/ERK/mTOR Pathway. PLoS One 9(11): e112186. 25372487 10.1371/journal.pone.0112186 PMC4221608

[15] Schmitter D, Lauber B, Fagg B, Stahel RA (1992) Hematopoietic growth factors secreted by seven human pleural mesothelioma cell lines: interleukin-6 production as a common feature. Int J Cancer 51: 296–301. 1373705 10.1002/ijc.2910510220

[16] Orengo AM, Spoletini L, Procopio A, Favoni RE, De Cupis A, Ardizzoni A, et al (1999) Establishment of four new mesothelioma cell lines: characterization by ultrastructural and immunophenotypic analysis. Eur Respir J 13: 527–534. 10232421 10.1183/09031936.99.13352799

[17] Workman P, Aboagye EO, Balkwill F, Balmain A, Bruder G, Chaplin DJ et al (2010). Guidelines for the welfare and use of animals in cancer research. British Journal of Cancer 102:1555–1577. 10.1038/sj.bjc.6605642 20502460 PMC2883160

[18] Muscella A, Vetrugno C, Antonaci G, Cossa LG, Marsigliante S (2015). PKC-δ/PKC-α activity balance regulates the lethal effects of cisplatin. Biochem Pharmacol 98: 29–40. 10.1016/j.bcp.2015.08.103 26300055

[19] Muscella A, Calabriso N, Vetrugno C, Fanizzi FP, De Pascali SA, Storelli C et al (2011) The platinum (II) complex [Pt(O,O'-acac)(g-acac)(DMS)] alters the intracellular calcium homeostasis in MCF-7 breast cancer cells, Biochem. Pharmacol. 81 91–103. 10.1016/j.bcp.2010.09.012 20854797

[20] Kindler HL (2008) Systemic treatments for mesothelioma: standard and novel. Curr Treat Options Oncol 9: 171–179. 10.1007/s11864-008-0071-3 18770046 PMC2782121

[21] Favoni R, Florio T (2011) Combined chemotherapy with cytotoxic and targeted compounds for the management of human malignant pleural mesothelioma. Trends Pharmacol Sci 32: 463–479. 10.1016/j.tips.2011.03.011 21620489

[22] Mujoomdar AA, Tilleman TR, Richards WG, Bueno R, Sugarbaker DJ (2010) Prevalence of in vitro chemotherapeutic drug resistance in primary malignant pleural mesothelioma: result in a cohort of 203 resection specimens. J Thorac Cardiovasc Surg 140: 352–355. 10.1016/j.jtcvs.2009.11.072 20653100

[23] Barbieri F, Würth R, Favoni RE, Pattarozzi A, Gatti M, Ratto A et al (2011) Receptor tyrosine kinase inhibitors and cytotoxic drugs affect pleural mesothelioma cell proliferation: insight into EGFR and ERK1/2 as antitumor targets Biochem Pharmacol 82: 1467–1477. 10.1016/j.bcp.2011.07.073 21787763

[24] Henkels KM, Turchi JJ (1999) Cisplatin-induced apoptosis proceeds by caspase-3-dependent and -independent pathways in cisplatin-resistant and -sensitive human ovarian cancer cell lines. Cancer Res 59: 3077–3083. 10397248

[25] Nowak G (2002) PKC-α and ERK1/2 mediate mitochondrial dysfunction, decreases in active Naþ transport, and cisplatin-induced apoptosis in renal cells. J Biol Chem 277: 43377–43388. 10.1074/jbc.M206373200 12218054 PMC1948818

[26] D’Amours D, Desnoyers S, D’Silva I, Poirier GG (1999) Poly(ADPribosyl)ation reactions in the regulation of nuclear functions. Biochem J 342: 249–268. 10455009 PMC1220459

[27] Bossy-Wetzel E, Newmeyer DD, Green DR (1998) Mitochondrial cytochrome c release in apoptosis occurs upstream of DEVD specific caspase activation and independently of mitochondrial transmembrane depolarisation. EMBO J 17: 37–49. 10.1093/emboj/17.1.37 9427739 PMC1170356

[28] Colussi PA, Harvey NL, Shearwin-Whyatt LM, Kumar S (1998) Conversion of procaspase-3 to an autoactivating caspase by fusion to the caspase-2 prodomain. J Biol Chem 273: 26566–26570. 9756894 10.1074/jbc.273.41.26566

[29] Germain M, Affar EB, D’Amours D, Dixit VM, Salvesen GS, Poirier GG (1999) Cleavage of automodified poly(ADP-ribose) polymerase during apoptosis. Evidence for involvement of caspase-7. J Biol Chem 274: 28379–28384. 10497198 10.1074/jbc.274.40.28379

[30] Kroemer G (1997) The proto-oncogene Bcl-2 and its role in regulating apoptosis. Nat Med 3: 614–620. 9176486 10.1038/nm0697-614

[31] Allen RT, Cluck MW, Agrawal DK (1998) Mechanisms controlling cellular suicide: role of Bcl-2 and caspases. Cell Mol Life Sci 54: 427–445. 10.1007/s000180050171 9645223 PMC11147391

[32] Narasimhan SR, Yang L, Gerwin BI, Broaddus VC (1998) Resistance of pleural mesothelioma cell lines to apoptosis: relation to expression of Bcl-2 and Bax. Am J Physiol 275: 165–171. 10.1152/ajplung.1998.275.1.L165 9688948

[33] Soini Y, Kinnula V, Kaarteenaho-Wiik R, Kurttila E, Linnainmaa K, Pääkkö P (1999) Apoptosis and expression of apoptosis regulating proteins bcl-2, mcl-1, bcl-X, and bax in malignant mesothelioma. Clin Cancer Res 5: 3508–3515. 10589765

[34] Hopkins-Donaldson S, Cathomas R, Simões-Wüst AP, Kurtz S, Belyanskaya L, Stahel RA et al (2003) Induction of apoptosis and chemosensitization of mesothelioma cells by Bcl-2 and Bcl-xL antisense treatment. Int J Cancer 106: 1660–1666. 10.1002/ijc.11209 12800189

[35] Emoto Y, Manome Y, Meinhardt G, Kisaki H, Kharbanda S, Robertson M et al (1995) Proteolytic activation of protein kinase C delta by an ICE-like protease in apoptotic cells. EMBO J 14: 6148–6156. 8557034 10.1002/j.1460-2075.1995.tb00305.x PMC394739

[36] Basu A, Woolard MD, Johnson CL (2001) Involvement of protein kinase C delta in DNA damage-induced apoptosis. Cell Death Differ 8: 899–908. 10.1038/sj.cdd.4400885 11526445

[37] Urso L, Muscella A, Calabriso N, Ciccarese A, Fanizzi FP, Migoni D et al (2005) Differential functions of PKC-delta and PKC-zeta in cisplatin response of normal and transformed thyroid cells. Biochem Biophys Res Commun 337: 297–305. 10.1016/j.bbrc.2005.09.046 16182242

[38] Fan M, Chambers TC (2001) Role of mitogen-activated protein kinases in the response of tumor cells to chemotherapy. Drug Res Updat 4: 253–267. 10.1054/drup.2001.0214 11991680

[39] Zhuang S, Demirs JT, Kochevar IE (2000) p38 mitogen-activated protein kinase mediates Bid cleavage, mitochondrial dysfunction, and caspase-3 activation during apoptosis induced by singlet oxygen, but not by hydrogen peroxide. J Biol Chem 275: 25935–25948. 10.1074/jbc.M001185200 10837470

[40] Galan A, García-Bermejo M L, Troyano A, Vilaboa NE, de Blas E, Kazanietz MG, et al (2000) Stimulation of p38 mitogen-activated protein kinase is an early regulatory event for the cadmium-induced apoptosis in human promonocytic cells. J Biol Chem 275: 11418–11424. 10753958 10.1074/jbc.275.15.11418

[41] Ko HW, Han KS, Kim EY, Ryu BR, Yoon WJ, Jung YK et al (2000) Synergetic activation of p38 mitogen-activated protein kinase and caspase-3-like proteases for execution of calyculin A-induced apoptosis but not N-methyl-d-aspartate-induced necrosis in mouse cortical neurons. J Neurochem 74: 2455–2461. 10820206 10.1046/j.1471-4159.2000.0742455.x

[42] Fennell DA, Rudd RM (2004) Defective core-apoptosis signalling in diffuse malignant pleural mesothelioma: opportunities for effective drug development. Lancet Oncol 5: 354–362. 10.1016/S1470-2045(04)01492-5 15172356

